# Epidemiological and Clinical Characteristics of Infections in Hospitalized Children During and After the COVID-19 Pandemic

**DOI:** 10.3390/v17101296

**Published:** 2025-09-24

**Authors:** Sandra Prgomet, Zvonimir Boban, Sunčica Prgomet, Nataša Boban

**Affiliations:** 1 Department of Pediatrics, University Hospital Centre Split, 21000 Split, Croatia; sandra.skember.prgomet@gmail.com; 2 Department of Medical Physics and Biophysics, University of Split School of Medicine, 21000 Split, Croatia; zvonimir.boban@mefst.hr; 3 Department of Mathematics, Faculty of Science, University of Split, 21000 Split, Croatia; suncica.prgomet1@gmail.com; 4 Department of Hospital Infections and Clinical Epidemiology, University Hospital Split, 21000 Split, Croatia; 5 Department of Public Health, University of Split School of Medicine, 21000 Split, Croatia

**Keywords:** COVID-19, children, respiratory viruses, enteric viruses, discharge diagnosis, presenting symptoms, non-pharmaceutical interventions

## Abstract

Infections, particularly those affecting the respiratory system, are a major cause of hospitalization among children. During the COVID-19 pandemic, the landscape of childhood infections underwent a significant transformation. To understand these changes, this study analyzes the epidemiological and clinical characteristics of infections in children hospitalized during the first quarters of years 2021–2024. The number of hospitalizations was four times greater in 2024 compared to 2021. The average patient age decreased from 4.6 years in 2021 to 2.3 years in 2024 due to the increase in proportion of infants. The most prevalent symptom changed from fever in 2021 to cough in subsequent years. Bacterial pathogens were dominant in 2021, and viral pathogens were more common in the other three years. SARS-CoV-2 and rotavirus were the most common viruses in 2021 and 2022 but were overtaken by influenza and respiratory syncytial virus in 2023 and 2024. The findings of the study highlight changes in patient characteristics caused by the easing of restrictions and subsequent resurgence of viral infections. Continued surveillance of infection trends is crucial for adapting clinical practices to the evolving challenges posed by infectious diseases in the post-pandemic world.

## 1. Introduction

The COVID-19 pandemic and the unprecedented public health measures implemented to mitigate its spread, including social distancing, mask-wearing, and school closures, profoundly impacted the dynamics of infectious diseases globally. Non-pharmaceutical interventions (NPIs), viral evolution, and immunity dynamics collectively reshaped pathogen circulation patterns, severity profiles, and long-term outcomes. Infections, particularly those affecting the respiratory system, are a major cause of hospitalization among children, and in over 80% are caused by viruses [1]. Understanding the epidemiology and clinical presentation of these infections is essential for developing and implementing effective prevention and treatment strategies.

While NPIs were effective at decreasing COVID-19 transmission, they simultaneously disrupted the epidemiological patterns of other respiratory viruses, particularly those affecting pediatric populations. Respiratory syncytial virus (RSV), influenza, and human rhinovirus—traditionally characterized by predictable seasonal circulation—experienced dramatic suppression during the initial phases of the pandemic, followed by irregular resurgence patterns as NPIs were relaxed [1,2]. Stringent NPIs during 2020–2021 reduced incidences of influenza, RSV, and enteroviruses by 50–90% across multiple countries [3,4]. Influenza detection rates dropped by 98% in New Zealand and RSV circulation was lowered to near-undetectable levels in Europe during initial lockdown periods [5,6,7]. In Bulgaria, RSV detections fell to 3.2% of pre-pandemic levels during 2020–2021, with similar declines observed for parainfluenza and human metapneumovirus [7,8]. Surveillance data from Turkey revealed a 78% reduction in RSV detection and a 92% decline in influenza positivity during peak NPI enforcement compared to pre-pandemic baselines [7]. Similar patterns emerged in South Africa, where facility-based surveillance documented near elimination of influenza and RSV from April to August 2020 [9]. These findings were corroborated by a multinational meta-analysis demonstrating a 67% lower RSV hospitalization risk during active NPI periods across 12 countries [2]. Aside from respiratory viruses, the incidence of common enteric viruses such as the rotavirus and adenovirus were also affected [10,11,12,13]. This unprecedented change was attributed to disrupted social mixing patterns that severed viral propagation chains, particularly for pathogens requiring close contact for transmission.

The aim of this study is to further elucidate the effects of the pandemic by analyzing the differences in the epidemiological and clinical characteristics of the causative agents of infections in hospitalized children during and after the COVID-19 pandemic.

## 2. Materials and Methods

### 2.1. Data Collection

The study was conducted in accordance with the Declaration of Helsinki, and approved by the Ethics Committee of the University Hospital of Split (No. 2181-147/01-06/LJ.Z.-25-02) on 30 April 2025. Data was collected from the hospital’s electronic health record system and anonymized to protect patient confidentiality. We recorded data of all children with a positive microbiological test. For every patient the type of sample, method of isolation of the microbiological agent and the type of isolate were collected. Microbiological isolates were identified using a range of diagnostic techniques, including cultures (for bacteria: coproculture, urinoculture, hemoculture), PCR (Multiplex PCR for detecting multiple respiratory pathogens in a single test using a nasopharyngeal swab) and rapid antigen tests for viruses, and serology (detection of IgM in the blood).

For the diagnosis of respiratory infections caused by adenoviruses, respiratory syncytial virus, Streptococcus pyogenes, influenza A and B, and SARS-CoV-2 virus, rapid tests based on the immunochromatographic method for detecting specific antigens were used, with samples obtained from nasopharyngeal swabs. In case of a negative rapid test and clinical indication, the nasopharyngeal swab was tested using Multiplex PCR (FILMARRAY) to simultaneously detect respiratory infection pathogens including Coronaviruses 229E, NL63, HKU1, and OC43; Human Metapneumovirus; Influenza A and B viruses; Middle East Respiratory Syndrome Coronavirus (MERS-CoV); Parainfluenza Viruses 1, 2, 3, and 4; Adenovirus; Human Rhinovirus/Enterovirus; Respiratory Syncytial Virus; *Bordetella parapertussis* (IS1001); *Bordetella pertussis* (ptxP); *Chlamydia pneumoniae*; and *Mycoplasma pneumoniae*. In the case of SARS-CoV-2 virus, if the rapid antigen test yielded a negative result despite high clinical suspicion, a confirmatory test was conducted using real-time reverse transcription polymerase chain reaction (rRT-PCR) for the detection of viral RNA.

For the diagnosis of intestinal infections, rapid immunochromatographic tests were used to detect antigens of intestinal adenoviruses, Helicobacter pylori bacteria, noroviruses, rotaviruses, and toxigenic Clostridium difficile bacteria. Stool samples were used for all these tests.

For other pathogens, bacterial infection diagnosis was performed by culturing microorganisms. Positive BacT/ALERT 3D blood cultures underwent Gram staining and was then inoculated onto the solid media for pathogen identification and direct antibiotic susceptibility testing. Subsequently, qualitative sensitivity testing was carried out, classifying bacteria as sensitive, intermediate, or resistant, with some antibiotics tested semi-quantitatively to determine minimum inhibitory concentration.

Signs and symptoms documented upon admission, included fever (defined as body temperature above 38 °C), cough, and symptom groups categorized as other respiratory (runny nose, nasal congestion, hoarseness, stridor, tachypnea, dyspnea, wheezing, chest retractions), neurological (loss or disturbance of consciousness, behavioral changes or hallucinations, facial palsy, headache, apnea, convulsions, hemiparesis), cardiovascular (chest pain, ECG changes, hypertension, palpitations/tachycardia), rheumatological (joint pain, muscle pain, joint swelling, skin rash), gastrointestinal (abdominal pain, nausea or loss of appetite, vomiting, diarrhea, weight loss or no weight gain, blood in stool), urinary (dysuria, hematuria, foul-smelling urine, renal colic, cloudy dialysate, leukocyturia), and other.

### 2.2. Data Analysis and Visualization

All data cleaning, data visualization and statistical analysis were performed using the R programming language version 4.4.2 [14]. The distribution normality of continuous variables was assessed using graphical inspection (histograms, Q-Q plots) and formal tests (Shapiro–Wilk or Kolmogorov–Smirnov). Categorical variables were analyzed using Pearson’s Chi-squared test to evaluate differences in proportions between groups. The Chi-squared goodness-of-fit test was applied to assess whether observed frequency distributions differed from those expected under the null hypothesis. For continuous or ordinal variables not meeting the assumptions of normality, differences between two independent groups were evaluated using the Mann–Whitney U test. Associations between continuous, approximately normally distributed variables were examined using Pearson’s product-moment correlation coefficient, with scatterplots used to visually inspect linearity. To assess the independent influence of predictor variables on binary outcomes, multivariable logistic regression models were constructed. Odds ratios with corresponding 95% confidence intervals were estimated. All statistical tests were two-sided, with a significance level set at α = 0.05. For each tested hypothesis, the statistical test and the obtained *p*-value were shown in parentheses in the results section.

## 3. Results

This retrospective study analyzes the medical data of all children aged 0–18 years, with positive microbiological finding, admitted to the Pediatric Clinic of the University hospital Split between 1 January and 31 March in 2021, 2022, 2023, and 2024. Trends in occurrence of various infectious agents, and their epidemiological and clinical characteristics were analyzed to examine similarities and differences between the patients hospitalized during the COVID and post-COVID period.

### 3.1. Patient Characteristics

**Figure 1:**
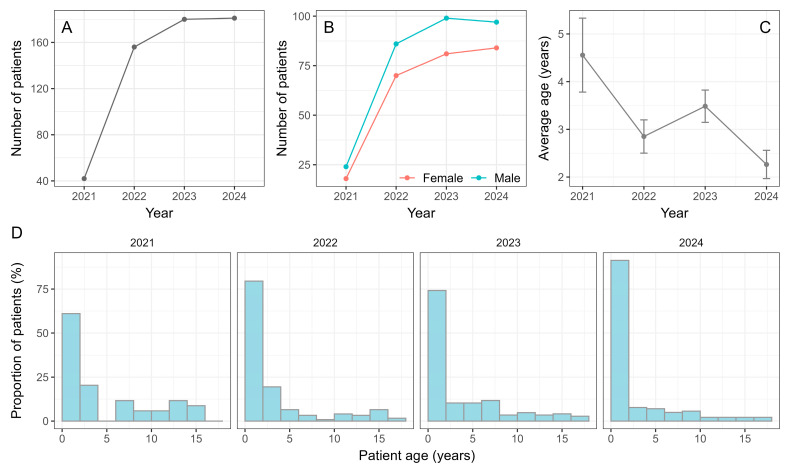
(**A**) The total number of admitted patients over the years. (**B**) The total number of admitted patients over the years with respect to patient sex. (**C**) The average age of admitted patients over the years. (**D**) The distribution of ages of admitted patients across the four years.

A total of 567 children were hospitalized with positive microbiological findings during the analyzed periods. The number of admitted patients was approximately four times greater in 2022 (n = 160) compared to the same period in 2021 (n = 43). Another smaller increase occurred from 2022 to 2023 when 182 hospitalizations were recorded, and then there was no change in the total number of admitted patients from 2023 to 2024 (n = 182) (Figure 1A). The change in the number of hospitalized patients over the years was statistically significant (*p* = 2.2 × 10^−16^, Chi-squared goodness of fit test).

The trend of the number of hospitalizations accounting for patient’s sex agreed with the overall trend very well, with male patients being slightly more numerous in all observed periods, although the difference was not statistically significant (*p* = 0.98, Pearson’s Chi-squared test) (Figure 1B).

Regarding the average age of patients, it was highest during 2021 at 4.6 years and then decreased by approximately 1.5 years in the following three periods (Figure 1C). An omnibus test showed a significant difference in the age between the four periods (*p* = 0.0081, Kruskal–Wallis rank sum test), with a significant difference between the 2021 and 2024 periods (*p* = 0.00043, Mann–Whitney test). The patient age distribution was positively skewed in all periods. The decrease in the average age in 2024 occurred due to a large increase in the proportion of infants (Figure 1D).

### 3.2. Presenting Symptoms

**Figure 2:**
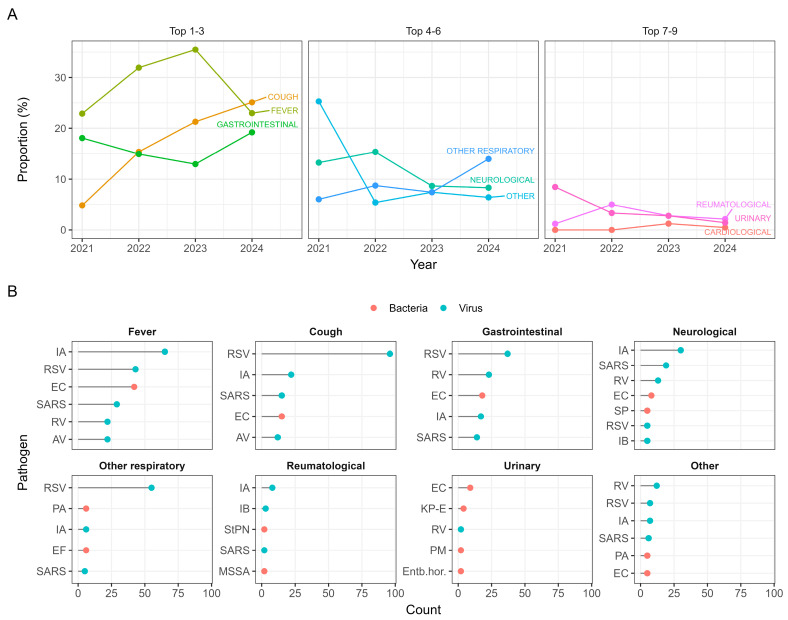
(**A**) The trends of proportions of the symptoms and signs across the four years. (**B**) The most frequent pathogens in each of the eight most common symptom categories.

The clinical presentation also differed across the observed years, with fever and cough being the most common ones (Figure 2A). Interestingly, while cough was the first or second most prevalent symptom during 2022–2024, practically no patients were admitted with cough symptoms during the first quarter of 2021. Figure 2B shows the most common pathogens associated with each symptom category. As expected, symptoms such as fever and cough are predominantly associated with viral infections, and urinary symptoms with bacterial infections.

**Figure 3:**
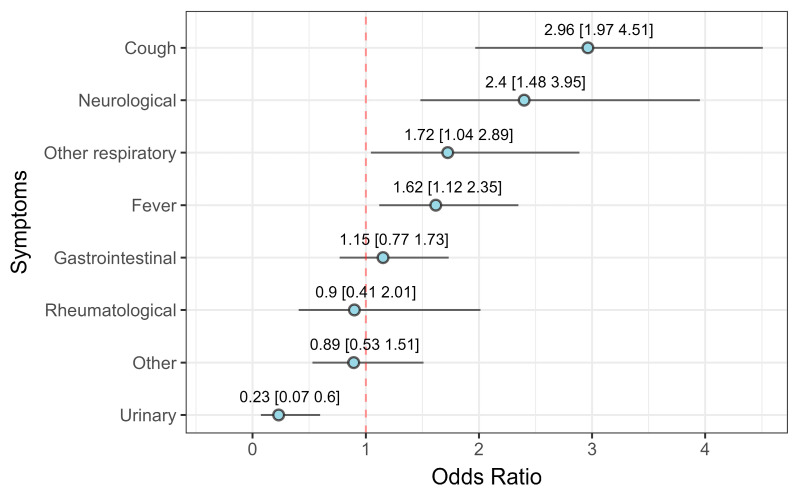
The odds ratio indicating the likelihood of detecting a viral pathogen. The horizontal lines denote the 95% confidence interval. The text above the lines shows the exact value of the odds ratio and its corresponding 95% confidence interval.

To quantify the likelihood of detecting a viral or bacterial pathogen depending on the symptoms and signs at admission, multivariable logistic regression was used to estimate the odds ratios for each symptom category (Figure 3). The results show that cough, neurological, other respiratory symptoms and fever were positively associated with viral pathogens, while displaying urinary symptoms increased the odds of an underlying bacterial infection. For other symptom categories, there was no statistically significant difference in association with either viral or bacterial pathogens.

### 3.3. Identified Pathogens

**Figure 4:**
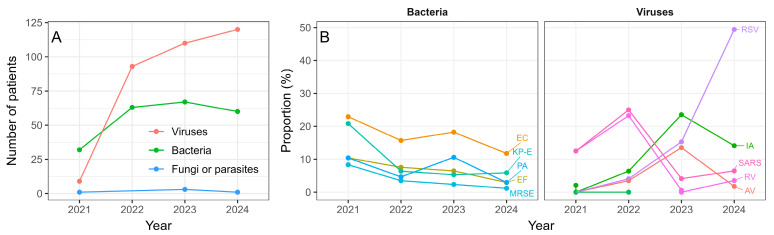
(**A**) The number of patients with a bacterial or viral isolate in each year. (**B**) The trend of occurrence of ten most common isolates overall throughout the years. Abbreviations: EC = *Escherichia coli*, KP-E = *Klebsiella pn. ESBL*, PA = *Pseudomonas aeruginosa*, EF = *Enterococcus faecalis*, RSV = Respiratory syncytial virus, IA = Influenza A, SARS = SARS-CoV-2 virus, RV = Rotavirus, AV = Adenovirus, IB = Influenza B.

The isolates’ profile changed over the years, with bacterial infections being more prevalent in 2021, and their proportion becoming lower than that of viral infections throughout 2022–2024. The number of infections due to fungi or parasites was negligible and practically constant over the years (Figure 4A, Table 1). The differences in proportions of patients with different pathogen types across the years were statistically significant (*p* = 2.2 × 10^−16^, Pearson’s Chi-squared test).

As expected, most common respiratory viral pathogens were mainly detected from nasopharyngeal samples, enteric viruses from stool samples, and bacteria from urine and blood samples (Table 2).

Observing the trends in occurrence of ten most common pathogens overall (Figure 4B), we can see that the proportion of most common bacterial isolates was highest in 2021, and then dropped to a stable value from 2022 to 2024, confirming the overall trend from Figure 4A. Conversely, the proportion of most common viral infections changed appreciably, with the SARS-CoV-2 and rotavirus being the most common in 2021 and 2022 and influenza and respiratory syncytial virus (RSV) increasing steeply and overtaking them in 2023 and 2024 (Figure 4B). There was a significant correlation between incidences of the SARS-CoV-2 and rotavirus isolates (r = 0.998, *p* = 0.0018, Pearson’s product-moment correlation).

### 3.4. Discharge Diagnoses

**Figure 5:**
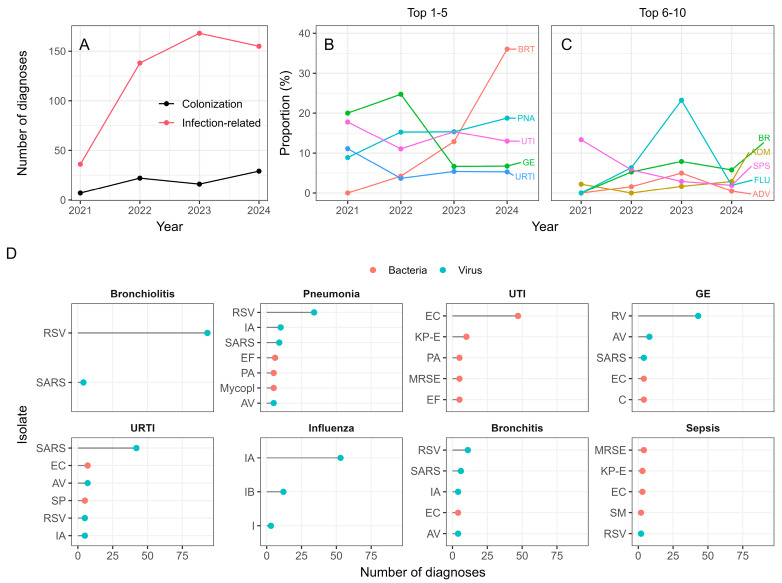
(**A**) The number of patients with and without an infection-related diagnosis at the time of discharge. (**B**,**C**) The trends of proportions of top ten most common diagnosis overall across the four years. (**D**) The most frequent isolates for eight most common discharge diagnoses. Abbreviations: BRT = bronchiolitis, PNA = pneumonia, UTI = urinary tract infection, GE = enteritis/gastroduodenitis, URTI = upper respiratory tract infection, FLU = influenza, BR = bronchitis, SPS = sepsis, ADV = adenovirus infection, AOM = acute otitis media.

Although the absolute number of hospitalized children increased over the years, the proportion of patients with an infection-related diagnosis remained stable with no statistically significant difference across the four periods (*p* = 0.19, Pearson’s Chi-squared test) (Figure 5A). Of the 567 hospitalized children, 468 (82.5%) received an infection-related diagnosis upon discharge. The proportion of patients with an infection diagnosis ranged from 70.3% in 2024 to 91.2% in 2023. In the remaining children, the microbiological isolate was considered more likely to be a colonization than the causative agent of the disease.

Figure 5B,C show the trend of top 10 most common diagnoses over the four analyzed periods. The enteritis/gastroduodenitis (GE) diagnosis was most common in 2021 and 2022. Although it did not occur at all in 2021 and displayed a relatively low incidence in 2022, the incidence of influenza increased suddenly in 2023, becoming the most common isolate in that year, and then dropped again in 2024. Increasing steadily from 2021, bronchiolitis became the most common diagnosis in 2024. Urinary tract infections (UTI) and pneumonias were among the top three diagnoses in all observed years. Upper respiratory tract infections (URTI) were the third most common diagnosis in 2021 when COVID was at its peak and then dropped to fifth place in the other three periods.

To inspect the relationship between the infection diagnoses and underlying pathogens, the most frequent underlying isolates were shown for the eight most commonly occurring infection diagnoses (Figure 5D). Bacterial isolates dominated the UTI and sepsis diagnoses, and viral isolates dominated the other six most common diagnoses (Figure 5D). The SARS-CoV-2 virus was most often associated with URTI and pneumonia diagnoses. Influenza was mostly caused by the IA virus, and less commonly by IB and other influenza types. The RSV was the most common underlying isolate in the case of pneumonia, bronchiolitis and bronchitis, followed by SARS-CoV-2.

Grouping pneumonia, bronchiolitis and bronchitis diagnoses together as lower respiratory tract infections (LRTI), it can be concluded that there was a significant difference in the frequency of different types of diagnoses related to SARS-CoV-2 (*p* = 4.54 × $10^{-8}$, Chi-squared test). SARS-CoV-2 was most often associated with URTI (48.8%), followed by LRTI (26.6%) and GE (4.7%) infections.

## 4. Discussion

The number of patients increased significantly from the first quarter of 2021 (n = 43) to the same period in 2024 (n = 182) (Figure 1A). This is consistent with other studies showing fewer pediatric hospitalizations in 2021 due to the impact of strict NPIs [15,16].

Furthermore, the parental healthcare-seeking behavior was also probably affected by the pandemic, as parents may have been more hesitant to bring their children to the hospital during the peak of the pandemic [17], underscoring the importance of considering the broader social context when interpreting epidemiological data.

The average patient age was higher in 2021 than in the other three years (Figure 1C,D). This most likely occurred due to the lower exposure of the youngest children to different social contacts and thus to different infectious agents, but also because of the low incidence of respiratory viruses, which in the most severe cases affect young children [1,2,7,18].

RSV is the leading cause of acute respiratory tract infections in young children, but infants younger than six months are at higher risk of severe disease and need for hospitalization [19,20]. This explains the highest proportion of the youngest patients in 2024 compared to other periods (Figure 1D), as there was a sharp increase in the number of RSV infections in 2024 (Table 1).

Easing of the restrictions in 2022 and 2023 resulted in a resurgence of infections, accompanied by a shift in the dominant pathogens and clinical presentations. The most prevalent presenting symptom changed from fever in 2021 to cough in subsequent years (Figure 2). This is in line with our results (Figure 3) and previous research, as the shift correlates with the resurgence of respiratory viral infections after the easing of restrictions (Table 1), and the presence of cough was shown to be more likely in the case of underlying viral pathogens compared to bacterial ones [21].

Regarding pathogens underlying the infections, they were dominantly bacterial in 2021, and then after the easing of NPIs in 2022, the viral pathogens took over as the most common type (Figure 4A). The initial suppression and subsequent rebound of respiratory viral infections are consistent with patterns observed globally [22]. Conversely, a relatively stable number of bacterial infections throughout the study period suggests a selective impact of COVID-19 prevention measures on the transmission dynamics of respiratory viruses. This finding indicates that while public health measures targeting respiratory droplet transmission may be effective in controlling viral infections, they may have a limited impact on bacterial pathogens spreading through different routes.

The relative ranking of the most common bacterial isolates remained similar throughout the observed period, but the ranking of most dominant viral pathogens changed each year (Figure 4B). SARS-CoV-2 and rotavirus were the most common isolates in 2021 and 2022, and influenza A and RSV during 2023 and 2024 (Figure 4B). Furthermore, apart from SARS-CoV-2, in 2021 and 2022 there was a negligible number of other common winter respiratory viruses like RSV and influenza A. These findings are corroborated by other studies showing a significant decline in RSV [2,9,23,24] and influenza [9,23,25,26]. The changes in respiratory virus epidemiology after the removal of NPIs could be attributed to the lack of immune stimulation by viruses during the pandemic [27,28].

Contrary to common respiratory viruses such as RSV and influenza, the occurrence of rotavirus was highly correlated with the occurrence of SARS-CoV-2, displaying a strong presence in both 2021 and 2022, but then practically disappearing in 2023 and 2024 (Figure 4B). The trend observed in the results agrees well with the results from other studies showing that the initial collateral benefit of coronavirus disease countermeasures that reduced the viral gastroenteritis burden is not sustainable, probably due to waning immunity and thus accumulation of susceptible population [11,12]. Furthermore, a study by Gunduz et al. showed that the number of emergency department visits due to rotavirus infections was higher during the COVID-19 period compared to the pre-pandemic period [29]. These results underline the need of accounting for the effect of the NPIs not only on transmission rates, but also on the proportion of susceptible population.

The total number of infection-related diagnoses was by far the lowest in 2021 while stricter measures were in place (Figure 5A). Bacteria related GE and UTI ranked highly in 2021 together with URTI caused by SARS-CoV-2. Once the measures were eased in 2022, there was an increase in the number of COVID and pneumonia cases, which correlates with the wave of infections and deaths from COVID in Q1 2022 [30]. After not being present at all in 2021, the flu returned in 2022, and the number of flu related hospitalizations exploded in 2023 (Figure 5B). The majority of children infected with Influenza viruses were diagnosed with URTI and mild to moderate bronchiolitis infections (Figure 5D), in agreement with a study by Rafeek et al. [31]. Reduction in bronchiolitis in 2021 and 2022 and its subsequent rise to the most common diagnosis in 2024 is directly correlated with the incidence of the RSV as its most common causative agent (Figure 5D). This is consistent with other studies showing that several NPIs were associated with a reduction in bronchiolitis outbreaks [32].

### Limitations

This study focuses on the first quarter of each year, providing a snapshot of infection trends during a period typically associated with higher respiratory infection rates. While this timeframe allows for targeted analysis, it limits the ability to assess seasonal variations across the entire year. This might be important in the context of the COVID-19 pandemic as several studies reported the recurrence of respiratory viruses out of season following the easing of containment measures [24]. Also, the data collection was limited to a single hospital, which may not represent the overall population. Future studies incorporating data from multiple hospitals and all four quarters would provide a more comprehensive understanding of seasonal patterns.

## 5. Conclusions

This retrospective study provides insights into the evolving landscape of pediatric infections during and after the COVID-19 pandemic in South Croatia. The findings underscore the effectiveness of public health measures in controlling the spread of respiratory viruses but also highlight the potential for rebound surges following the easing of restrictions. Continued surveillance of infection trends and a thorough understanding of the interplay between viral and bacterial infections are crucial for informing effective public health strategies and adapting clinical practices to the evolving challenges posed by infectious diseases in the post-pandemic world.

## Figures and Tables

**Table 1:** Incidence of most common pathogens over the four observed periods.

Pathogen	Abbreviation	2021	2022	2023	2024	Total
**Viruses**						
Respiratory syncytial virus	RSV	-	6	26	74	106
Influenza A	IA	-	11	39	24	74
SARS-CoV-2	SARS	6	36	4	9	55
Rotavirus	RV	4	35	-	5	44
Adenovirus	AV	-	5	22	1	28
Influenza B	IB	-	-	11	1	12
**Bacteria**						
*Escherichia coli*	EC	7	18	23	16	64
*Enterococcus faecalis*	EF	3	9	3	2	17
*Klebsiella pn. ESBL*	KP-E	3	7	1	5	16
*Pseudomonas aeruginosa*	PA	1	4	6	2	13
*Methicillin-resistant Staphylococcus epidermidis*	MRSE	3	3	2	1	9
*Bordetella bronchiseptica*	Bordetella	-	-	-	6	6
Mycoplasma	Mycopl	1	-	-	4	5
Strept.pyogenes	SP	1	-	2	2	5

**Table 2:** Percentages of sample types used for detecting the eight most common pathogens across four most common sampling methods.

Pathogen	Abbreviation	Nasopharyngeal	Stool	Blood	Urine
**Viruses**					
Respiratory syncytial virus	RSV	100	-	-	-
Influenza A + B	IA + IB	98.2	1.1	-	-
SARS-CoV-2	SARS	98.9	-	1.8	-
Adenovirus	AV	67.9	25	3.6	-
Rotavirus	RV	-	97.7	-	-
**Bacteria**					
*Escherichia coli*	EC	-	-	-	98.4
*Enterococcus faecalis*	EF	-	-	11.8	82.4
*Klebsiella pn. ESBL*	KP-E	-	-	12.5	87.5

## Data Availability

Dataset available on request from the authors.
